# The Hidden Impact of Gestational Diabetes: Unveiling Offspring Complications and Long-Term Effects

**DOI:** 10.3390/life15030440

**Published:** 2025-03-11

**Authors:** Elsa Al Bekai, Carla El Beaini, Karim Kalout, Ouhaila Safieddine, Sandra Semaan, François Sahyoun, Hilda E. Ghadieh, Sami Azar, Amjad Kanaan, Frederic Harb

**Affiliations:** 1 Faculty of Medicine and Medical Sciences, University of Balamand, Kalhat, Tripoli P.O. Box 100, Lebanonhilda.ghadieh@balamand.edu.lb (H.E.G.);; 2 Family & Geriatric Medicine, Centre Hospitalier du Nord–CHN, Zgharta P.O. Box 100, Lebanon; 3 AUB Diabetes, American University of Beirut Medical Center, Beirut P.O. Box 11-0236, Lebanon

**Keywords:** gestational diabetes mellitus (GDM), maternal hyperglycemia, fetal hyperinsulinemia, neonatal hypoglycemia, metabolic syndrome, neurodevelopmental disorders, developmental origins of health and disease (DOHaD) theory

## Abstract

Background: Gestational diabetes mellitus (GDM), characterized by gestational hyperglycemia due to insufficient insulin response, poses significant risks to both maternal and offspring health. Fetal exposure to maternal hyperglycemia leads to short-term complications such as macrosomia and neonatal hypoglycemia and long-term risks including obesity, metabolic syndrome, cardiovascular dysfunction, and type 2 diabetes. The Developmental Origins of Health and Disease (DOHaD) theory explains how maternal hyperglycemia alters fetal programming, increasing susceptibility to metabolic disorders later in life. Objective: This review explores the intergenerational impact of GDM, linking maternal hyperglycemia to lifelong metabolic, cardiovascular, and neurodevelopmental risks via epigenetic and microbiome alterations. It integrates the most recent findings, contrasts diagnostic methods, and offers clinical strategies for early intervention and prevention. Methods: A comprehensive literature search was conducted in PubMed, Scopus, and ScienceDirect to identify relevant studies published between 1 January 2000 and 31 December 2024. The search included studies focusing on the metabolic and developmental consequences of GDM exposure in offspring, as well as potential mechanisms such as epigenetic alterations and gut microbiota dysbiosis. Studies examining preventive strategies and management approaches were also included. Key Findings: Maternal hyperglycemia leads to long-term metabolic changes in offspring, with epigenetic modifications and gut microbiota alterations playing key roles. GDM-exposed children face increased risks of obesity, glucose intolerance, and cardiovascular diseases. Early screening and monitoring are crucial for risk reduction. Practical Implications: Understanding the intergenerational effects of GDM has important clinical implications for prenatal and postnatal care. Early detection, lifestyle interventions, and targeted postnatal surveillance are essential for reducing long-term health risks in offspring. These findings emphasize the importance of comprehensive maternal healthcare strategies to improve long-term outcomes for both mothers and their children.

## 1. Introduction

Gestational diabetes mellitus (GDM) is defined as gestational hyperglycemia as a result of an insufficient insulin response diagnosed during pregnancy [1,2,3]. It is thought that the high blood glucose levels in pregnant women are due to insulin resistance caused by the hormone human placental lactogen (hPL) [4]. GDM is reported to be associated with many short-term and long-term complications postpartum [5], the most important of which is the increased risk of developing type 2 diabetes mellitus later in life [2]. There is no consensus on how to diagnose diabetes in pregnancy, but the most agreed upon diagnostic technique is to use the two-step testing approach starting with screening with a GCT proceeding diagnosis with a 100 g, 3-h oral glucose tolerance test (OGTT) for those who screen positive. However, the 2018 guidelines of the American College of Obstetrician and Gynecologists (ACOG) acknowledge the use of a one-step approach using 1 and 2 h fasting glucose values for a 75 g, 2-h OGTT if this method is appropriate for the tested population [2,6]. It is usually diagnosed during the second and third trimesters [5]. GDM can be classified into two main categories: A1GDM, which can be managed through diet without medication, and A2GDM, which requires medication to regulate blood glucose levels during pregnancy [7].

The prevalence of GDM varies depending on the diagnostic criteria used [6]. Regardless, the numbers are rising internationally, and this is directly correlated to the increased levels of obesity over a long-time frame [3]. The prevalence of GDM among pregnant women increased from 1.2% to 2.3% between 1976 and 2010, coinciding with the observed increase in obesity levels in the US [2]. Recently, the percentage of diagnosed women with GDM in the United States increased from 6.0% to 8.3% in the period between 2016 and 2021, and the percentage of diagnosed patients increased in a direct correlation with age [2].

In addition to the negative consequences that gestational diabetes mellitus might impose on the mother [8], it is also associated with multiple complications in the offspring [5]. These complications include metabolic disorders, which comprise obesity, adiposity [9], and impaired glucose tolerance [9]. Moreover, it affects the cardiovascular, renal, and gastrointestinal systems, as it increases the risks for hypertension [10], nephropathy [11], and nonalcoholic fatty liver disease [12], respectively. The complications of gestational diabetes mellitus (GDM) may impact the neurodevelopment of the infant and contribute to macrosomia through distinct mechanisms [13]. Additionally, GDM complications might increase the risk of osteoporosis [14], hyperlipidemia [15], and infertility in the offspring [11]. Therefore, it is crucial to develop techniques that would allow the early diagnosis of GDM in pregnant women so that early management would be possible in order to prevent the development of the aforementioned complications in the offspring of a pregnant woman with GDM [16]. Therefore, the aim of this review is to uniquely examine the intergenerational impact of GDM, integrating the DOHaD theory to explain how maternal hyperglycemia causes long-term metabolic, cardiovascular, and neurodevelopmental complications in offspring. It highlights epigenetic modifications, transgenerational inheritance, and gut microbiome alterations, linking prenatal GDM exposure to lifelong disease risk. Additionally, this review provides an updated synthesis of the new recent findings, contrasts diagnostic methods, and offers clinical recommendations for early screening and intervention to prevent intergenerational disease transmission.

## 2. Methods and Literature Search Strategy

To conduct this literature review, we performed a comprehensive search across multiple databases, including PubMed, Cochrane Library, EMBASE, Google Scholar, and other relevant academic repositories. Our search strategy focused on identifying peer-reviewed studies published between 1 January 2000 and 31 December 2024, covering topics related to gestational diabetes mellitus (GDM) and its short- and long-term effects on offspring. We used a combination of MeSH terms and keywords, such as gestational diabetes mellitus, fetal programming, metabolic syndrome, epigenetics, neurodevelopmental disorders, and offspring health outcomes. The search was refined using filters to include systematic reviews, meta-analyses, cohort studies, and randomized controlled trials, ensuring that only high-quality and relevant literature was selected.

To ensure the inclusion of seminal and recent findings, we manually screened the titles and abstracts of all retrieved articles. Duplicates were removed, and full-text reviews were conducted to assess methodological quality, relevance, and consistency with our research objectives. References of key articles were also reviewed to identify additional studies that met our inclusion criteria. Studies focusing on diagnostic methods, fetal metabolic adaptations, the developmental origins of health and disease (DOHaD) theory, and maternal–fetal interactions were prioritized. This rigorous search strategy allowed us to synthesize a comprehensive, evidence-based review of the intergenerational impact of GDM, providing valuable insights into preventive strategies, clinical management, and long-term health implications.

## 3. Fetal Programming and Developmental Origins of Health and Disease (DOHaD)

### 3.1. Perinatal Milieu and the Long-Term Developmental Programming of Progeny

GDM is a glucose tolerance condition that first appears during pregnancy [17]. During pregnancy, higher amounts of hormones like prolactin, estrogen, and lactogen are produced. This helps shift nutrients from the mother to the fetus and prevents low blood glucose by blocking insulin effects. Insulin resistance emerges in the middle of gestation and increases as gestation progresses. In an attempt to offset the diabetogenic impact of placental hormones, maternal pancreatic islets attempt to increase insulin production by up to three times during pregnancy. When maternal β-cells cannot adjust to increased fetal nutrient requirements and increasing insulin needs during late pregnancy, blood glucose levels increase, spurring the fetal pancreas to produce more insulin [18]. Over time, exposure to GDM may increase both the maternal and fetal risk of acquiring non-communicable diseases (NCDs). NCDs are a significant public health concern and are frequently viewed as diseases of adult lifestyle. They most likely have a complex etiology with many risk pathways and interactions between genetic and environmental variables [19].

### 3.2. Developmental Origins of Health and Disease

**Figure 1:**
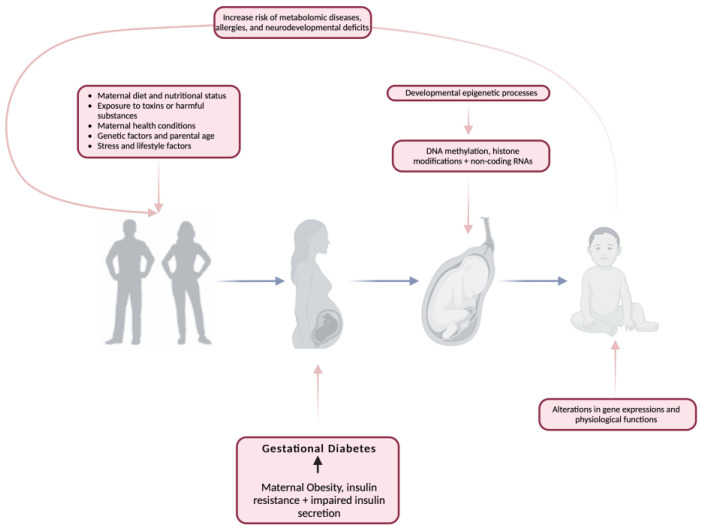
The impact of gestational diabetes mellitus on developmental programming. Created with https://www.biorender.com (accessed on the 12 June 2024).

The period from conception to early infancy plays a crucial role in shaping long-term health outcomes, with early-life factors such as nutrition and environmental exposures influencing disease susceptibility later in life [19,20,21,22]. This concept, known as developmental programming, suggests that the conditions experienced in utero and shortly after birth can have lasting effects on metabolic health. Gluckman et al. [21] highlight the intricate differences between developmental modeling influenced by prenatal and postnatal exposures, making it challenging to separate their individual contributions (Figure 1). However, both perspectives converge on the idea that disruptions during early development—whether through altered growth patterns or metabolic programming—can significantly increase the risk of NCDs such as obesity, type 2 diabetes, and cardiovascular disease [23,24,25,26,27].

The first 1000 days after conception are particularly critical, as they set the foundation for lifelong health. During this period, epigenetic modifications and metabolic reprogramming may occur, potentially predisposing individuals to chronic conditions. By identifying biomarkers at birth and closely monitoring key developmental windows, early intervention strategies can be implemented to reduce disease risk and improve long-term health outcomes. This proactive approach could not only prevent the onset of metabolic disorders but also lessen the economic burden on healthcare systems by reducing the need for later treatment [21].

Barker’s seminal research laid the foundation for the metabolic programming theory, demonstrating that individuals with low birth weight face a higher risk of developing cardiovascular disease, hypertension, type 2 diabetes, and metabolic syndrome later in life [28]. Human cohort studies and animal experiments further support this, showing that fetal undernutrition during critical stages of organ and metabolic system development—such as the pancreas, heart, kidney, liver, skeletal muscle, and endocrine axes—can lead to permanent structural and functional alterations, increasing susceptibility to cardio-metabolic diseases in adulthood [29,30,31,32]. Simpson’s study reinforces this concept, emphasizing that the intrauterine environment plays a key role in shaping lifelong health trajectories. The intrauterine environment exposes the fetus to what life could be “ex-utero” despite the potential disparity between the two. Fetal exposure to maternal over- or undernutrition, epigenetic modifications, and environmental stressors can create a metabolic “memory”, leading to maladaptive changes in organ morphology and function. This developmental plasticity not only increases the risk of cardiovascular and metabolic dysfunction but also facilitates the intergenerational transmission of disease, particularly in cases of maternal obesity. Poor maternal health and nutrition during pregnancy may set the stage for long-term health challenges in offspring, perpetuating a cycle of chronic disease across generations. These findings highlight the need for targeted prenatal interventions to mitigate early-life metabolic programming and reduce the burden of non-communicable diseases in future generations. On the other hand, developmental overnutrition is supported by the DOHaD theory as a mechanism for transmitting metabolic dysfunction to subsequent generations. This is particularly relevant with rising maternal obesity and maternal diabetes. In a “healthy” pregnancy, blood glucose levels rise, and moderate insulin resistance develops in order to satisfy the needs of the growing fetus [32]. A genuine association persists between the mother’s blood glucose levels and the birth weight of her offspring [33]. This risk also persists into childhood, as maternal glucose concentration is reflective of childhood adiposity [34] and insulin resistance independently of maternal body mass index (BMI) [35].

### 3.3. Developmental Programming by Epigenetic Mechanisms

**Figure 2:**
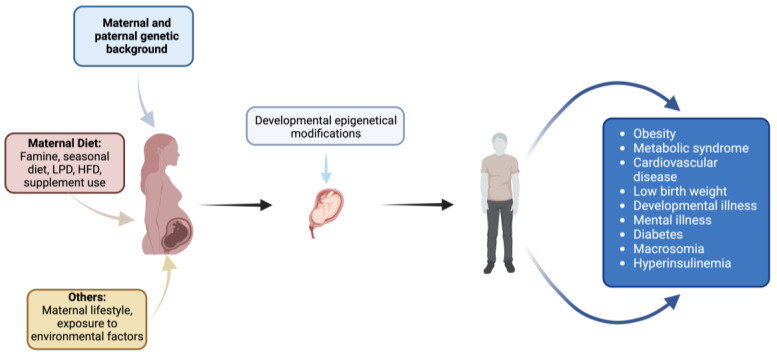
Impact of maternal factors on fetal development and offspring health: environmental, dietary, genetic, and lifestyle influences. Created with https://www.biorender.com (accessed on the 1 April 2024).

Within the framework of fetal programming, epigenetic mechanisms play a crucial role in determining long-term consequences for offspring [36]. These mechanisms regulate gene expression and genomic structure without altering DNA sequences and are specific to certain cell types and tissues. Key epigenetic processes include DNA methylation, histone post-translational modifications, and non-coding RNA expression (Figure 2).

GDM has been implicated in influencing preterm birth through epigenetic modifications, particularly DNA methylation. Studies have shown that maternal hyperglycemia can lead to altered DNA methylation patterns in the offspring’s cord blood. For instance, in one study, maternal GDM was associated with lower cord blood methylation levels within two regions, including the promoter of *OR2L13*, a gene associated with autism spectrum disorder, and the gene body of *CYP2E1*, which is upregulated in type 1 and type 2 diabetes [37].

Furthermore, research indicates that maternal diabetes during pregnancy can cause DNA hypermethylation at various individual CpG sites and regions, which has been linked to an increased risk of preterm delivery [38]. Identifying DNA methylation patterns linked to adverse health outcomes in offspring may serve as an early biomarker for individuals at risk, offering a critical window for early intervention during childhood [19].

A strong association exists between in utero hyperglycemic exposure and altered cord blood DNA methylation patterns [38]. Maternal diabetes during pregnancy has been linked to DNA hypermethylation at various CpG sites and regions, which may contribute to an increased risk of preterm delivery [38]. Additionally, differentially methylated positions (DMPs) during pregnancy have been correlated with preterm birth risk [38]. This aligns with previous findings demonstrating a positive association between gestational diabetes and preterm birth [39], defined as delivery before 37 weeks of pregnancy [40]. However, the increased incidence of preterm birth in women with GDM may also be influenced by healthcare providers recommending early delivery to mitigate complications associated with fetal overgrowth [39].

Polyhydramnios, characterized by excessive accumulation of amniotic fluid during pregnancy, is another complication commonly associated with GDM and can also induce preterm delivery [41]. Furthermore, GDM has been linked to a higher incidence of cesarean section (C-section) [42], primarily due to fetal overgrowth resulting from maternal hyperglycemia. This condition often necessitates a C-section to prevent complications during labor and vaginal delivery [41].

### 3.4. Transgenerational Epigenetic Inheritance

According to Chu and Goddfrey, growing evidence from animal models—mostly involving mice and rats—indicates that developmental programming occurs transgenerationally. Several potential processes could result in the transmission of the programmed phenotype, such as the inheritance of epigenetic modifications through modification of the epigenome (germline and/or somatic line), altered maternal phenotype, and persistence of harmful environmental exposures in subsequent generations [19]. A pattern of dysregulation at critical methylation sites in the placenta was observed in a GDM mice model of intrauterine hyperglycemia caused by streptozotocin. This was indicated by downregulation of the *Dlk1* (gene involved in cell growth and differentiation during development and associated with metabolism and adipogenesis) and overexpression of the *Gtl2* (gene implicated in various cellular processes and diseases, including cancer) genes in the F1 and F2 generations, respectively [43].

A study carried out in The Gambia revealed that seasonal exposure to hunger is associated with altered methylation of the pro-opiomelanocortin (POMC) gene, which helps regulate body weight [44]. The peri-conceptual stage is emphasized in this study as a crucial time for epigenetic modification and developmental flexibility. Children conceived during the rainy or “hungry” season, when nutrition was less abundant, showed higher levels of POMC gene methylation, higher BMIs, and an increased risk of obesity compared to those conceived during the dry season, when nutrition was more abundant [44].

Although the transgenerational transfer of characteristics has been documented up to the F2 offspring, there is still conflicting evidence about the transmission to the F3 and later generations [45]. Despite compelling evidence that epigenetic alterations are passed down across generations in mice and rats, some have questioned the applicability of this idea to humans [46]. This comes as a result of a complicated combination of numerous confounding factors, such as ecological and cultural inheritance [47].

## 4. Offspring Complications Associated with Maternal GDM

### 4.1. Macrosomia and Neonatal Complications

One of the most immediate concerns related to the offspring of a mother with GDM is fetal macrosomia. The prevalence of fetal hyperinsulinemia resulting from maternal hyperglycemia increases the risk of macrosomia in newborns, with 15–45% of macrosomic babies born to mothers with GDM [16]. Women with GDM have a 2–3 times greater risk of delivering macrosomic babies in comparison to women with normal pregnancy [48]. Maternal hyperglycemia, as a result of GDM, leads to an increase in maternal insulin resistance and subsequently causes an increase in glucose transfer to the fetus through the placenta [49]. The excess glucose absorbed by the fetus is stored as body fat and in turn leads to the development of a baby that is large for gestational age (LGA) [49].

Macrosomia refers to newborns with a birth weight ≥4000 g or above the 90th percentile for gestational age. Among infants of mothers with GDM, 15–45% develop macrosomia [16], compared to 12% in non-GDM pregnancies, increasing their risk of obesity and type 2 diabetes. Contributing factors include maternal obesity, gestational age at delivery, pre-pregnancy BMI, weight gain, hypertension, and smoking [49]. Studies indicate that fetal birth weight is most strongly associated with second- and third-trimester postprandial blood glucose levels rather than fasting glucose levels, with a threshold of ≤120 mg/dL correlating to a 20% incidence of macrosomia [49]. If the value of glucose is as high as 160 mg/dL, then the rate of macrosomia increases to 35%. The pathophysiology of macrosomia is best understood using Pedersen’s hypothesis, which elucidates the ways in which maternal hyperglycemia leads to fetal hyperinsulinemia and elevated fetal adipose tissue. Since glucose can freely cross the placenta, while insulin cannot, in the second trimester the fetus’s pancreas begins secreting its own insulin in response to the elevated fetal glucose levels. The combination of fetal hyperglycemia and hyperinsulinemia leads to increased fat and protein storage, resulting in macrosomia. These neonates are at heightened risk for hypoglycemia, which can lead to central nervous system and cardiopulmonary complications, including mental retardation, recurrent seizures, developmental delay, and personality disorders [49].

Macrosomic infants of GDM mothers often exhibit a central deposition of subcutaneous fat, particularly in the abdomen and interscapular areas, along with increased shoulder and extremity circumferences but a normal head size. This growth pattern is associated with an increased risk of shoulder dystocia (SD), a complication that occurs when the infant’s shoulder becomes lodged behind the symphysis pubis, potentially leading to neonatal brachial plexus injury, asphyxia, and postpartum hemorrhage [50]. The incidence rate of SD is significantly higher among macrosomic infants and those born via vacuum-assisted delivery, occurring at a rate of 196.7 per 1000 births compared to 19.5 per 1000 in the absence of these risk factors [50].

In addition to SD, macrosomia increases the risk of birth trauma, including Erb’s palsy, clavicle fractures, and brachial plexus injuries, often necessitating neonatal intensive care unit (NICU) admission. The Australian Carbohydrate Intolerance Study in Pregnant Women (ACHOIS) found a strong association between maternal fasting hyperglycemia severity and SD risk, with each 1-millimole increase in fasting glucose correlating to a 2.09-fold increased risk of SD [49]. Macrosomic newborns weighing ≥4500 g are six times more likely to experience birth trauma and twenty times more likely to suffer brachial plexus injuries. Compared to infants of non-GDM mothers, macrosomic neonates have a five-fold increased risk of severe hypoglycemia and a two-fold increase in neonatal jaundice.

Macrosomia is also linked to increased risk of preterm birth, respiratory difficulties, feeding issues, infections, and perinatal mortality. The hyperinsulinemic environment of macrosomic infants drives an increased oxygen demand, triggering erythropoietin (EPO) secretion and resulting in polycythemia [51]. This condition can lead to neonatal jaundice due to excessive bilirubin from red blood cell breakdown [49]. Additional congenital anomalies linked to macrosomia include heart defects and neural tube defects such as spina bifida [49].

### 4.2. Neonatal Hypoglycemia and Metabolic Effects

Neonatal hypoglycemia is one of the most common metabolic disorders in infants of GDM mothers. A prospective cohort study defined severe neonatal hypoglycemia as blood glucose ≤36 mg/dL and mild hypoglycemia as ≤47 mg/dL, with risk factors including macrosomia, prematurity, maternal diabetes, and low birth weight for gestational age. Among 2145 pregnant women screened at 24–28 weeks, GDM was diagnosed in 583, with neonatal hypoglycemia observed in 33.4% of cases and severe hypoglycemia in 20.2% [52]. Another study on 597 pregnant women with GDM found that 39% of newborns had hypoglycemia, with the highest incidence in neonates whose mothers were treated with glyburide, reinforcing the need for careful glucose management to mitigate risks [53].

Maternal hyperglycemia, caused by GDM, induces fetal hyperinsulinemia and in turn leads to the accelerated fetal reuptake of glucose through the placenta [54]. The excess fetal reuptake of glucose results in the overgrowth of fetal adipose tissue and can be identified by the increase in offspring fat mass (FM), percent fat mass (%FM), and skinfold thickness [54]. Studies have found no significant difference in total adipose tissue volume at birth between children born to mothers with GDM or those born to normoglycemic mothers [55]. However, by the second to third months postnatally, this difference in total adiposity became notably significant, with infants of mothers with GDM exhibiting a 16% increase in total adipose tissue volume compared to the control group [55]. According to this study, the strict management of glucose levels of GDM mothers during pregnancy might have contributed to the weak variances in neonatal adiposity outcomes between the group of newborns to mothers with GDM and the control group [55]. GDM infants showed rapid weight gain, and this could be explained by hypothalamic alteration in hunger and satiety cues as a result of the prolonged hyperglycemic exposure of GDM infants in utero [55]. 

### 4.3. Long-Term Health Risks: Obesity, Metabolic Syndrome, and Cardiovascular Disease

High birth weight has been linked to a higher risk of obesity and higher body fat percentage during childhood and adolescence [56]. Studies have shown a positive correlation between maternal hyperglycemia and an increase in total fat percentage in children aged between 10 and 14 years old [16], whereas, in children of East Asian descent, BMI values were remarkably analogous between children born to mothers with GDM and those born to normoglycemic mothers (16.0 kg/m^2^ and 16.1 kg/m^2^) [16]. A body composition analysis revealed a positive correlation between maternal glucose levels and an increase in total fat mass in children born to mothers with GDM, irrespective of their ethnicities [16]. In summary, employing body composition analysis is advantageous for assessing the risk of adiposity in children born to mothers with GDM, regardless of their ethnic backgrounds [16].

Obesity in childhood and even adulthood is another complication that has become linked to pregnancies with GDM. The majority of newborns with mothers who have GDM are born larger than average, and numerous studies have shown that this excess weight remains throughout life, leading to overweight or obesity. This increase in BMI has been proven to cause adverse complications such as diabetes mellitus type 2, cardiovascular diseases, metabolic syndrome, and even cancers. A multi-ethnic study conducted to assess the association between exposure to maternal diabetes in utero and BMI growth trajectories showed that a significant increase in BMI growth velocity was observed among children between the ages of 10 and 13. In this time frame, children who were exposed to diabetes in utero experienced a 4.56 kg/m^2^ increase, compared with a 3.51 kg/m^2^ increase in unexposed children (*p* = 0.005) [57]. Additionally, this study showed no significant difference in growth trajectories between the two groups during infancy and early childhood [57]. In comparison, another study proved that GDM diagnosed using the WHO 1999 [58] criteria was independently associated with an increased BMI in children ages 1 through 6 [59]. Such inconsistencies may be due to the changes in the diagnostic criteria of GDM, small sample size, or various other factors such as cross-sectional study design and residual confounding [60]. On average, the number of women diagnosed with GDM increased two-fold in 2014 when new diagnostic criteria were set by the IADPSG as compared to the previous criteria set by the World Health Organization or the American Diabetes Association [61].

A BMI ≥ 85th percentile but <95th percentile was defined as childhood overweight, while a BMI ≥ 95th percentile was defined as obesity [61]. In a study conducted on 10,412 mother–child pairs, with 17.2% involving GDM cases, it was concluded that the increased BMI Z score and overweight/obesity at ages 2 to 4 were primarily influenced by the pre-pregnancy BMI of the mother rather than direct GDM exposure [60]. Other factors that influence the risk of obesity in children born to mothers with GDM include breastfeeding and lifestyle habits. Studies have shown a 22% decrease in the risk of developing childhood obesity in breastfed infants compared to those who have never been breastfed [62]. However, more extensive research must be conducted regarding this correlation. A longitudinal study conducted on children exposed to GDM and type 1 diabetes in utero aimed to compare the difference between diabetic breastmilk and nondiabetic banked donor breastmilk intake during the first week following delivery and its effect on body weight at age 2 [63]. The results of the study showed an increased body weight in children consuming diabetic breast milk compared to those who were fed banked donor breast milk, and this effect was only observed during the first week of life since no association was found between drinking diabetic breast milk during weeks 2 to 4 and body weight at age 2 [63]. These findings suggest that the initial week of life may represent a crucial period for the nutritional programming of offspring born to mothers with GDM.

It is common for a child whose mother had GDM to be overweight or obese. Pediatric obesity, in itself, augments several cardiovascular risk factors such as high blood pressure, abnormal lipid profile, impaired glucose tolerance, and metabolic syndrome [64]. This was further evident in a meta-analysis and systematic review conducted by Umer et al., which revealed a complex relationship between childhood adiposity and adult blood pressure. Childhood adiposity was positively associated with adult systolic blood pressure (SBP) when unadjusted for adult adiposity; however, when adjusted for adult adiposity, the association became significantly negative. A similar trend was observed for diastolic blood pressure (DBP), where an initial positive correlation was seen with childhood adiposity, but when adjusted for adult adiposity, the association turned negative [64].

Another factor that plays a critical role in the development of obesity in GDM-exposed children is the duration of breastfeeding. Compared to infants breastfed for less than 3 months, those who were breastfed for more than 3 months had a 45% risk reduction of being overweight at ages 2 to 8 years [65]. Additionally, a study examining the relationship between GDM exposure and eating behaviors in adolescents, using the Eating in the Absence of Hunger in Children and Adolescents (EAH-C) questionnaire, found a positive correlation between the two factors. It was proposed that GDM-exposed children exhibit dysfunctional hunger and satiety signaling, potentially leading to food consumption in the absence of hunger, thereby increasing adiposity and excess body weight. Notably, offspring of GDM pregnancies are overnourished in utero [66]. This study also found significantly higher EAH-C scores in females exposed to GDM in utero compared to males, with females also reporting higher scores due to fatigue and/or boredom compared to males. Overall, the study suggests that the increased risk of obesity in GDM-exposed children may be driven by increased caloric intake and altered hunger signaling mechanisms [66].

The metabolic consequences of GDM exposure extend beyond childhood eating behaviors and obesity risk. Neonatal hypoglycemia is considered as one of the most common fetal complications of GDM pregnancies [67]. Studies demonstrated that fetal hyperinsulinemia resulting from exposure to hyperglycemia in utero can lead to hypoglycemia in the newborn at birth [49]. The persistence of fetal hypoglycemia postnatally can be harmful and raises serious concerns over its long-term effect on the baby’s neurological development, leading to mental retardation, recurrent seizures, and developmental delay [49]. The occurrence of fetal hypoglycemia is correlated with birth weight, term birth, improper feeding, and maternal GDM [67]. Therefore, preterm babies who are born to mothers with GDM are at a higher risk of developing hyperinsulinism-related hypoglycemia [67].

For many years, the uterine environment was believed to be sterile, with the first microbial colonization thought to occur during vaginal delivery. However, bacterial DNA detected in the meconium of newborns challenged this notion [68]. Consequently, the role of gut microbiota in mediating GDM complications has been explored. Studies have shown that neonates born to mothers with GDM exhibit a significantly lower level of operational taxonomic units (OTUs) compared to those born to non-GDM mothers. Specifically, GDM-exposed neonates had alterations in gut microbiota composition, including the enrichment of *Faecalibacterium* species and depletion of key genera such as *Veillonella*, *Megasphaera*, *Prevotella*, and *Rothia* [69]. These findings suggest that maternal GDM influences early gut microbial colonization, which may have implications for neonatal and long-term metabolic health.

Apart from the increased risk of obesity and diabetes, children born to mothers with GDM are prone to the development of metabolic syndrome (MetS), which is a collection of metabolic abnormalities typically characterized as three or more of the following: central obesity, reduced high-density lipoprotein cholesterol, hypertriglyceridemia, hyperglycemia, and hypertension. Like GDM, MetS is also associated with an increase in the risk of developing chronic diseases such as type 2 diabetes mellitus and cardiovascular disease (CVD). A recent meta-analysis showed that in utero exposure to GDM resulted in a significantly increased risk of developing MetS later in life (RR 2.07, 95% CI 1.26–3.42; three studies, 4421 participants; heterogeneity I^2^ = 12%; *p* = 0.33), a two-fold increase compared to offspring not exposed to GDM [70]. It has been proposed that the increased risk is due to a GDM-induced hyperinsulinemic environment, resulting in macrosomia and future obesity. In addition to the increased risk of MetS on offspring, there is an increased risk of developing MetS in pregnant women suffering from GDM, either during their pregnancy or at a later stage, typically one year postpartum [70].

There is a robust association between hyperglycemic disorders during pregnancy and fetal cardiovascular alterations. A study conducted on 264 mother–child pairs, including 116 cases where the mothers had GDM, identified several notable differences between children born to mothers with GDM and those born to healthy mothers. For instance, children born to mothers with GDM were shown to have a thicker posterior left ventricular wall but a significantly lower end systolic left ventricular volume. Additionally, they had significantly smaller aortic valve ejection time and pulmonary valve maximal velocity compared to children born to mothers without GDM [71]. Another study showed that these children were more prone to developing a variety of valvular defects [72].

### 4.4. Neurodevelopmental Disorders and Gut Microbiota Alterations

In addition to all the physical implications of GDM on offspring, recent studies have been conducted to identify the association between GDM and neurodevelopmental disorders such as autism spectrum disorder (ASD) and attention-deficit hyperactivity disorder (ADHD). Both ASD and ADHD are common neurodevelopmental disorders, often diagnosed in childhood. ASD encompasses a range of conditions such as impaired communication and repetitive behaviors, while ADHD is characterized by impulsivity, hyperactivity, and the inability to remain focused. Both disorders heavily impact an individual’s quality of life, leading to struggles in both personal and professional life. A meta-analysis and systematic review found that children under 18 exposed to GDM in utero had a 42% increased risk of developing ASD (pooled OR 1.42, 95% CI 1.22–1.65), while no significant association was observed for ADHD (pooled OR 1.01, 95% CI 0.79–1.28) [73]. This elevation could be attributed to either the selection of at-risk samples or the systematic measurement of symptoms. Compared to the general population, the median prevalence in those exposed to GDM was higher, with ASD having a median prevalence of 16.3% and ADHD 14.4% [74,75]. Considerable heterogeneity was observed among these studies, as shown by the wide IQRs for these medians. However, since the baseline risk of the development of neurobehavioral disorders is low in the general population, the relative risk of developing ASD or ADHD in an offspring born to a mother with GDM is also relatively low [73]. To provide a clearer understanding of the complications in offspring associated with maternal GDM, we have summarized all available information in Table 1.

## 5. Clinical Implications on Management

GDM is an early indicator of type 2 diabetes, creating a disrupted intrauterine environment that profoundly affects fetal development. Maternal hyperglycemia exposes the fetus to abnormal glucose levels, increasing the likelihood of both low and high birth weights, each carrying distinct long-term metabolic risks. Low birth weight, often a result of placental insufficiency, is linked to insulin resistance and a heightened susceptibility to metabolic syndrome, while high birth weight (macrosomia) is associated with an increased risk of childhood obesity, type 2 diabetes, and cardiovascular disease [29].

Given these risks, pregnancy serves as a critical window for intervention, offering a unique opportunity to implement preventative measures against type 2 diabetes for both the mother and child. By incorporating early screening, nutritional guidance, and structured physical activity, maternal glucose metabolism can be improved, reducing excessive fetal exposure to glucose and mitigating metabolic dysfunction in offspring [76]. Moreover, targeted prenatal interventions can play a pivotal role in breaking the cycle of intergenerational diabetes transmission, improving long-term health outcomes.

With the rapid rise in maternal obesity and sedentary lifestyles, the urgency for these interventions has never been greater. Poor maternal health behaviors contribute not only to excessive fetal growth but also to metabolic reprogramming, predisposing offspring to chronic diseases that manifest well into adulthood. Pregnancy presents a prime opportunity to counteract these effects by promoting healthy dietary habits and physical activity, which can prevent the downward spiral toward metabolic disease. Addressing these risk factors through prenatal and postpartum care could significantly reduce the global diabetes burden and enhance overall public health [77,78].

### 5.1. Screening and Diagnosis of GDM

Screening for GDM is crucial, as it allows for the early identification, timely intervention, and prevention of serious maternal and fetal complications [79]. Screening helps detect GDM in asymptomatic pregnant women, enabling proactive management [16]. The International Association of the Diabetes and Pregnancy Study Groups (IADPSG) developed clinical criteria for GDM screening, which includes a glucose challenge test (GCT). This involves administering 50 g of glucose and measuring serum glucose levels after one hour, without fasting. A result of ≥130–140 mg/dL is considered positive, requiring confirmation with an OGTT [16]. Many countries implement this two-step screening approach to achieve early diagnosis, particularly in the first trimester [80]. In Spain, adopting this method led to an increased incidence of GDM diagnoses, yet reduced adverse pregnancy outcomes due to earlier intervention [16]. However, while this approach helped treat more women, it did not significantly decrease the incidence of LGA births [81].

Another approach for the early screening of GDM involves measuring glycosylated hemoglobin (HbA1c) levels [82]. Although its use as a GDM screening marker remains controversial [83], HbA1c has been suggested as a promising early indicator of GDM [84]. It is favored over other screening methods due to its ease of measurement and potential for early prediction [85]. However, HbA1c alone has low sensitivity for diagnosing GDM [86]. An HbA1c level >5.4% suggests an increased risk of developing GDM, warranting an OGTT for confirmation [87]. Elevated HbA1c levels in early pregnancy correlate with higher risks of fetal complications [88]. Conversely, first-trimester HbA1c (Ft-HbA1c) levels <5.2% classify pregnant women as low risk, eliminating the need for additional testing [83]. Even if early HbA1c screening appears normal, it is recommended to repeat screening in the third trimester, particularly if initial levels were near the borderline range [85]. While HbA1c is mainly a screening marker, some studies propose HbA1c ≥ 5.5% as a diagnostic threshold when combined with other risk factors [89].

### 5.2. Preferred Methods for GDM Screening and Diagnosis

The selection of the most appropriate screening method depends on the individual risk profile of the pregnant woman [90]. If a woman presents with multiple risk factors for GDM, HbA1c screening is preferred due to its early detectability and ease of use [91,92,93,94]. However, while the GCT is widely used, its low accuracy prevents it from replacing the OGTT, which remains the gold standard for definitive GDM diagnosis [94]. Similarly, HbA1c alone is insufficient for diagnosis, but when used in combination with other clinical indicators, it can contribute to GDM detection [91]. Furthermore, incorporating HbA1c screening may reduce the number of OGTT tests required, thereby lowering healthcare costs [92].

### 5.3. Monitoring GDM

Once GDM is diagnosed, it is essential to monitor glucose levels to prevent maternal–fetal complications [95]. One of the most effective monitoring tools is continuous glucose monitoring (CGM), which allows for real-time glucose tracking and facilitates early intervention through lifestyle modifications or pharmacotherapy [96]. Studies show that CGM helps regulate maternal weight gain and reduces fetal macrosomia postpartum [95]. Although capillary blood glucose (CBG) measurement is also used, its effectiveness is limited to a narrow range of complications. For example, CBG monitoring alone has not been shown to reduce the incidence of macrosomia [97].

There are additional monitoring methods aimed at reducing postpartum complications associated with GDM. One of the most common is ultrasound, which is recommended for mothers with GDM to assess fetal growth and identify macrosomia or LGA fetuses [98,99]. Some studies suggest that high ultrasonographic fetal biomarkers (>90th percentile) during the second trimester may indicate an increased risk of developing pregnancy-related complications, including GDM, although these biomarkers are not exclusive to GDM [100].

Another important prenatal monitoring tool is non-stress testing (NST), which evaluates fetal well-being by assessing fetal heart rate patterns in response to movement [101]. NST is widely used in GDM-complicated pregnancies to reduce neonatal complications and determine the optimal delivery timing [102,103,104]. A reactive NST, characterized by fetal heart rate acceleration in response to movement, indicates a lower risk of fetal distress and stillbirth due to maternal hyperglycemia exposure [101].

### 5.4. Interventions for Managing GDM

**Figure 3:**
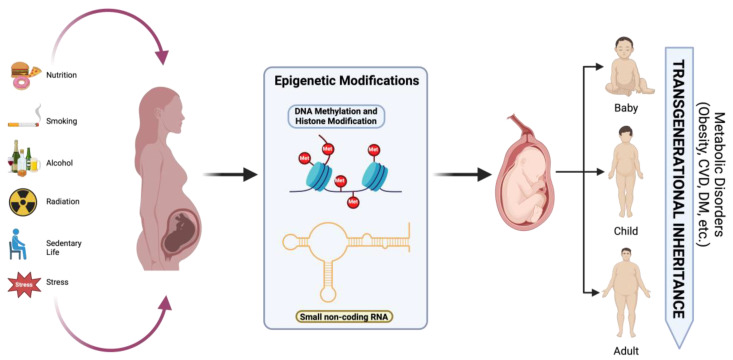
Effects of nutrition, hyperglycemia, smoking, radiation, psychological stress, and alcohol consumption on epigenetic modifications and long-term metabolic disorders in offspring. Created with https://www.biorender.com (accessed on the 21 April 2024).

To prevent maternal and neonatal complications, patients with GDM undergo various interventions, including medical nutritional therapy (MNT) and pharmacological treatment [2] (Figure 3). Approximately 80–90% of patients are managed with MNT alone, which helps reduce macrosomia and neonatal adiposity, although achieving postpartum weight goals remains challenging [105]. MNT emphasizes balanced meals rich in whole grains, proteins, and unsaturated fats to maintain a low glycemic index (GI) [106]. Some studies suggest that increasing protein intake from plant-based sources may be more beneficial than from red meat, as it helps improve insulin sensitivity [5].

In addition, probiotic supplementation has been explored as a means of improving glycemic control and reducing the incidence of GDM [107]. Probiotics restore gut microbiota balance, normalize intestinal permeability, and modulate pro-inflammatory markers, thereby lowering glycemic levels and improving lipid metabolism [108,109]. Meal planning is also critical in managing postprandial glucose spikes, and bedtime snacking has been shown to prevent starvation ketosis that may develop during overnight fasting [2].

Pairing MNT with physical activity is essential, with moderate aerobic exercise of 30 min per session, at least five days per week, totaling 150 min per week being recommended [110]. However, any consistent form of physical activity, regardless of duration, is beneficial for women with GDM [111].

When MNT alone is insufficient, pharmacological therapy is implemented. Insulin is the preferred medication, as it does not cross the placenta and can be tailored to patient needs [2]. Depending on gestational age and insulin resistance levels, dosages are adjusted within a range of 0.7–1 unit per kg [2]. Long-acting or intermediate-acting insulin may be combined with rapid-acting insulin based on glycemic requirements [2].

Finally, oral hypoglycemic agents, such as metformin and glyburide, are generally not recommended. Metformin crosses the placenta and may result in fetal concentrations equal to or higher than maternal levels [112]. While metformin improves insulin action, reduces gluconeogenesis, and lowers pregnancy-induced hypertension, it is also associated with higher rates of preterm birth [112,113]. Glyburide use has been linked to neonatal complications, including macrosomia and neonatal hypoglycemia [114].

## 6. Conclusions

GDM is associated with multiple fetal complications, including fetal hyperinsulinemia, neonatal hypoglycemia, macrosomia, which is associated with polycythemia, and delivery complications such as shoulder dystocia. GDM increases the risk of preterm delivery through multiple mechanisms, including epigenetic changes, fetal overgrowth, and excess amniotic fluid, all of which can necessitate early medical intervention. Finally, GDM correlates strongly with increased birth weights and rapid weight gain later in life, possibly from hypothalamic alterations, leading to obesity.

GDM increases the risk of both the mother and offspring acquiring non-communicable diseases. This is determined by developmental programming, which indicates that early intrauterine events have a major impact on developing diseases later in life. Nutritional deficiencies during pregnancy can predispose the offspring to glucose intolerance later in life, regardless of when the deficiency occurs. However, if the deficiency happens during the first trimester, it specifically increases the risk of developing cardiovascular diseases in the future. In addition, maternal obesity and diabetes may lead to placental insufficiency, which may result in either fetal growth restriction or overgrowth. Fetal overgrowth predisposes to both childhood and adult obesity. Many of these complications seem to be mediated via epigenetic mechanisms and modifications supporting long-term consequences in the progeny.

Screening and monitoring for GDM is important in the early identification and management of the disease. This involves the glucose challenge test, Hb1Ac, continuous glucose monitoring, ultrasound, and prenatal non-stress testing. However, definitive diagnosis is through the oral glucose tolerance test.

There exist some interventions to mitigate the GDM effect on infants. These include medical nutritional therapy, the use of probiotics, and if needed, pharmacological therapy. Insulin infusion is the gold standard, as it does not cross the placenta. Oral agents can be used but they are not recommended because glyburides have other adverse effects on the infant, and metformin can cross the placenta and reach the infant. Incretins can act directly or indirectly through activating insulin, but their effect is minimal.

Management of potential complication due to macrosomia entails delivery through cesarean section to prevent shoulder dystocia. Neonatal hypoglycemia can be managed by decreasing the mother’s glucose levels intrapartum. Hyperbilirubinemia can be treated with phototherapy and non-invasive respiratory support in the NICU for NRDS and infants with bronchopulmonary dysplasia. As for hypocalcemia, IV calcium infusion can be used in acute phases, and vitamin D or calcium supplements in chronic hypocalcemia. Preterm birth infants with feeding problems are managed through continuous breastfeeding and feeding modifications. Childhood obesity is managed prenatally through monitoring of the mother’s diet and pharmacological treatment and postpartum through altering the newborn’s diet, with the Mediterranean diet being recommended. Routine physical activity is recommended to reduce the risk of developing metabolic syndrome.

Research on the complications of GDM on both the mother and offspring has yielded several positive outcomes with regards to screening and diagnosis, which have significantly improved in the early identification of women with GDM, leading to timely intervention. This has greatly improved neonatal pregnancy outcomes and promoted long-term health for GDM women [115]. Improvement in patient counseling has played a large role, with healthcare providers discussing the risks and the healthy lifestyles to be implemented to reduce the risks of GDM. Self-management postpartum is possible, with a healthier lifestyle preventing diabetes after pregnancy [116]. Being a risk factor for cardio-metabolic disease [117], the approach to GDM requires an integrative approach into addressing the long-term offspring complications [6].

Understanding GDM and its complications has many implications in public health. It allows the healthcare system to implement preventative measures to reduce the incidence of GDM and its associated complications. This may be accomplished in several ways, as simple as promoting healthy lifestyles [118,119] and increasing awareness about and access to prenatal care [120,121]. This is achieved by the deployment of policies aiming at providing prenatal healthcare to underserved populations. One example was constructing a public health-based endocrine specialty clinic, which helped in providing healthcare to low-income pregnant women with GDM [122]. It is important to raise awareness on the importance of early detection and the risks of developing GDM. This awareness should address both pregnant women and healthcare providers through health education [123].

In conclusion, GDM is a complex and potentially preventable condition with significant short- and long-term sequelae. It is essential to address this implication by applying proactive measures that aim at improving the health and well-being of the mother with GDM and her fetus. Improving early detection and management is the key to preventing any complications. It is important to merge the efforts of healthcare practitioners, policymakers, and basic researchers culminating in better health outcomes for the pregnant mother, fetus, newborn, and future adult.

## Figures and Tables

**Table 1:** Summary of studies on offspring complications associated with maternal GDM.

**Author Name**	**Year**	**Study Design**	Sample Characteristics	Complications Discussed	Findings	Limitations
Moon et al. [16]	2022	Review	Not applicable	GDM-related maternal and offspring complications	Summarizes diagnostic approaches and maternal–offspring complications of GDM	Lacks primary data; relies on existing literature
Blasi et al. [48]	2023	Observational	Women with GDM	Fetal growth, neonatal hypoglycemia, and hyperbilirubinemia	Identifies correlations between glycemic variability and neonatal outcomes	Potential confounders not fully controlled
Kc et al. [49]	2015	Literature review	Not applicable	Macrosomia	Summarizes associations between GDM and macrosomia	Does not provide new data
Voormolen et al. [52]	2018	Cohort study	Pregnant women with GDM	Neonatal hypoglycemia	Finds higher neonatal hypoglycemia risk in GDM pregnancies	Limited generalizability due to sample size
Kole et al. [53]	2020	Observational	Neonates of GDM mothers	Neonatal hypoglycemia	Identifies factors associated with neonatal hypoglycemia	Retrospective design may introduce bias
Logan et al. [55]	2016	Cohort study	Infants of mothers with GDM	Early adiposity	Finds increased adiposity in infants exposed to GDM	Lack of long-term follow-up
Crume et al. [57]	2011	Longitudinal cohort	Children exposed to maternal diabetes	BMI growth trajectories	Finds increased BMI growth velocity in exposed children	Potential for unmeasured confounders
Wang et al. [59]	2019	Cohort study	Children aged 0–6 years	Offspring growth	Finds higher BMI in GDM-exposed children	Limited ethnic diversity
Shi et al. [60]	2020	Cohort study	Children aged 1–4 years	Offspring BMI	Links maternal GDM to increased BMI in young children	Cross-sectional analysis limits causal inference
Yan et al. [62]	2014	Meta-analysis	Breastfeeding and childhood obesity	Childhood obesity	Finds breastfeeding reduces obesity risk	Heterogeneity among included studies
Plagemann et al. [63]	2002	Longitudinal study	Children of diabetic mothers	Neonatal breastfeeding and metabolic outcomes	Finds neonatal breastfeeding impacts body weight and glucose tolerance	Small sample size
Shapiro et al. [66]	2017	Observational	Adolescents exposed to maternal diabetes	Eating behavior	Links maternal diabetes to altered eating behaviors	Self-reported data may introduce bias
Zhao et al. [67]	2020	Observational	Neonates	Neonatal hypoglycemia	Identifies risk effectors for neonatal hypoglycemia	Single-center study limits generalizability
Crusell et al. [69]	2020	Comparative study	Offspring of mothers with and without GDM	Gut microbiota	Finds differences in gut microbiota composition in GDM-exposed neonates	Lack of longitudinal follow-up
Pathirana et al. [70]	2021	Meta-analysis	Women with GDM and offspring	Metabolic syndrome	Finds increased metabolic syndrome risk in GDM-exposed offspring	Heterogeneity among included studies
Hromadnikova et al. [72]	2020	Molecular study	Children of GDM mothers	Diabetes and cardiovascular risk	Finds altered microRNA expression in GDM-exposed offspring	Needs clinical validation
Rowland et al. [73]	2021	Meta-analysis	Children of mothers with GDM	ASD and ADHD	Finds GDM exposure increases ASD risk but not ADHD	Potential publication bias
